# Design of a retrospective patient record study on the occurrence of adverse events among patients in Dutch hospitals

**DOI:** 10.1186/1472-6963-7-27

**Published:** 2007-02-25

**Authors:** Marieke Zegers, Martine C de Bruijne, Cordula Wagner, Peter P Groenewegen, Roelof Waaijman, Gerrit van der Wal

**Affiliations:** 1 NIVEL, Netherlands Institute for Health Services Research, PO Box 1568, 3500 BN Utrecht, The Netherlands; 2 EMGO Institute, VU University Medical Centre (VUmc), Van der Boechorststraat 7, 1081 BT Amsterdam, The Netherlands

## Abstract

**Background:**

Various international studies have shown that a substantial number of patients suffer from injuries or even die as a result of care delivered in hospitals. The occurrence of injuries among patients caused by health care management in Dutch hospitals has never been studied systematically. Therefore, an epidemiological study was initiated to determine the incidence, type and impact of adverse events among discharged and deceased patients in Dutch hospitals.

**Methods/Design:**

Three stage retrospective patient record review study in 21 hospitals of 8400 patient records of discharged or deceased patients in 2004. The records were reviewed by trained nurses and physicians between August 2005 and October 2006. In addition to the determination of presence, the degree of preventability, and causes of adverse events, also location, timing, classification, and most responsible specialty of the adverse events were measured. Moreover, patient and admission characteristics and the quality of the patient records were recorded.

**Discussion:**

In this paper we report on the design of the retrospective patient record study on the occurrence of adverse events in Dutch hospitals. Attention is paid to the strengths and limitations of the study design. Furthermore, alterations made in the original research protocol in comparison with former international studies are described in detail.

## Background

Various studies have shown that a substantial number of patients suffer from injuries or even die as a result of care delivered in hospitals [1-11]. The studies revealed that 2.9% to 16.6% of patients in acute care hospitals experienced one or more adverse events and that in 5% to 13% of the adverse events patients died. Approximately 50% of the adverse events were considered as preventable. An adverse event is defined as an unintended injury that results in disability at the time of discharge, death, or prolonged hospital stay and is caused by health care management rather than by the patient's underlying disease process [1,3,9,11]. The large variation in the incidence of adverse events among the studies in different countries may either be explained by true differences in patient safety of the different health care systems, or by methodological differences between the studies. Therefore, extrapolation of results from other countries will not give a valid estimate of patient safety in health care in e.g. Dutch hospitals.

The occurrence of injuries among patients caused by health care management in Dutch hospitals has never been studied systematically. Analysis of complaints, complications, medical errors and incidents recorded in Dutch hospitals and taken from data on claims and disciplinary proceedings, has shown – not surprisingly – that adverse events are occurring in Dutch hospitals [12-14] However, such data are inappropriate to estimate the incidence of adverse events, because completeness of the registrations depends on the willingness to report in the hospitals and a standardisation of the registration systems is lacking. Therefore, in 2005, as first part of the Dutch Patient Safety Research Program, a study was initiated to determine the incidence, nature, type, impact and costs of adverse events among hospitalised patients in the Netherlands. Insight in preventable adverse events can offer a starting point for specific interventions to improve patient safety in hospitals.

The method used in this study was based on a protocol originally developed by the Harvard Medical Practice Study, which has studied the incidence of adverse events in New York state hospitals in 1984, based on analysis of information in patient records [2]. This protocol, with modifications, was used in subsequent studies in Australia, the United Kingdom, New Zealand, the United States (in Colorado and Utah), Denmark, France and Canada [1-3,6,8-11,15] The protocol and instruments used in this Dutch study are based on the most recent retrospective study of adverse events in hospitals, which was carried out in Canada. A pilot study showed that the method and instruments, with some modifications, were valid and appropriate for the study of adverse events in Dutch hospitals [16]. However, our pilot study showed moderate to poor inter-rater reliability in the determination of adverse events and their preventability. Also in the previous studies inter-rater reliability appeared to be a major problem [1-3,9,11,17-19] Therefore, standing on the shoulders of our predecessors and keeping the method and instruments maximally comparable, we have tried to improve the reliability of the adverse events determination.

The retrospective patient record study is the first epidemiological study on the occurrence of adverse events in Dutch hospitals. The objectives of this study are to (1) determine the incidence, nature, type, impact, and costs of adverse events among hospitalised discharged and deceased patients; (2) examine the causes and preventability of these adverse events; (3) compare the rate of adverse events and preventable deaths between hospital types and between main specialties; and (4) compare the Dutch incidence of adverse events in acute care hospitals with international rates.

In this paper we report on the design of the retrospective patient record study on the occurrence of adverse events in Dutch hospitals. Attention is paid to the strengths and limitations of the study design. Furthermore, alterations made in the original research protocol in comparison with former international studies are described in detail. The results will be published in a separate article.

## Methods/Design

### Design and setting

The study is a retrospective patient record study carried out in Dutch hospitals. Patient records of randomly selected admissions of patients discharged in 2004 and admissions of patients who died in the hospital in 2004 were reviewed in a three stage review process by nurses and physicians between August 2005 and October 2006.

### Definitions

The definitions used in this study were adopted from previous adverse event studies to enable a comparison of the results of this study with previous international studies. [1,3,9,11]. The definitions are mentioned in table 1. Table 2 gives examples of cases with and without adverse events and preventability.

**Table 1:** Definitions

An **adverse event **is an unintended injury that results in temporary or permanent disability, death or prolonged hospital stay and that is caused by health care management rather than by the patient's underlying disease process.
**Unintended Injury **refers to any disadvantage for the patient that leads to prolonged or strengthened treatment, temporary or permanent (physical or mental) impairment or death.
**Disability **refers to temporary or permanent impairment of physical or mental function attributable to the adverse event (including prolonged or strengthened treatment, prolonged hospital stay, readmission, subsequent hospitalisation, extra outpatient department consultations or death).
**Causation **refers to injury caused by health care management including acts of omission (inactions) i.e. failure to diagnose or treat, and acts of commission (affirmative actions) i.e. incorrect diagnosis or treatment, or poor performance.
**Health Care management **includes the actions of individual hospital staff as well as the broader systems and care processes. Health care management is any care related activity that involves the delivery of care or monitoring of health which is provided by individuals or a team of professionals.
**Preventable adverse event **is an adverse event resulting from an error in management due to failure to follow accepted practice at an individual or system level. Accepted practice was taken to be 'the current level of expected performance for the average practitioner or system that manages the condition in question'.

**Table 2:** Examples of cases with and without adverse events and preventability

***No Adverse event (outcome of disease) [25]***
An 80-year-old man presented with a myocardial infarction with three hours of chest pain. He was treated promptly with streptokinase, heparin and aspirin. On day 3 he had further chest pain, with new ECG changes, and he died 12 hours later of cardiogenic shock.
***Adverse event (no preventability) [11]***
A 50-year old woman underwent coronary angiography for unstable angina. During the angiogram she sustained an anaphylactic reaction to the contrast, with cardiac arrest. She was able to be resuscitated promptly, without permanent sequelae, and hospitalisation was prolonged by 10 days. Evidence for prior contrast reactions was sought and not found.
***Adverse event (no preventability) [1]***
Abdominal pain and fever following elective surgical procedure. Patient readmitted for antibiotic treatment.
***Adverse events (low preventable) [25]***
Young right handed man sustained a fracture of the radius within the wrist joint. It required operative reduction, K-wire fraction and bone grafting. At the 10-day check the position had shifted and re-operation was required. The end result was very good.
***Adverse event (high preventability) [11]***
A 67-year old woman underwent a laparoscopic cholecystectomy, which proceeded to an open operation. Endoscopic retrograde cholangiopancreatography was undertaken eight days after the operation to remove a gallstone in the common bile duct; cannulation was not possible and the procedure was aborted. Ten days after the operation the patient collapsed and died suddenly. Autopsy findings showed extensive deep venous thrombosis and saddle pulmonary embolus. There was no documented evidence of thromboembolic prophylaxis in the medical record.
***Adverse event (high preventability) [1]***
Admission because of severe anaemia. The anaemia had been documented in previous admission but not investigated fully, which resulted in delayed diagnosis of colorectal carcinoma.

### Power calculation

The selection of hospital admissions was stratified for admissions of patients discharged alive and admissions of patients who died in the hospital. In patients who died during admission, the incidence of preventable adverse events associated with the death of the patient was assessed. Moreover, the incidence of adverse events was expected to be higher in this group, which made the study more efficient.

The power calculation of this study was based on the results of the Canadian adverse event study [1]. Assuming an incidence of adverse events during hospital admission of 8%, a sample of 4200 hospital admissions of discharged patients and a sample of 4200 admissions of deceased patients were necessary to detect an one-side difference of 1% with the reference value with a power of 0.80 and an alpha from 0.05. Because of difference in patient mix and delivery of care, we expected a difference in incidence between hospitals. Therefore, the incidence per hospital type was measured. To measure the difference in incidence between hospital types a selection of 800 hospital admissions per hospital type were necessary to detect a difference from 2% to 3% by an incidence between 3% and 7%.

### Hospital selection

To determine a national wide adverse events incidence rate a random sample of 21 hospitals, which is 20% of the hospitals in the Netherlands, was selected following stratification by hospital type and geographical area taken the density of population per region into account. The selected hospitals were: 4 university hospitals (out of 8), 6 tertiary medical teaching hospitals (out of 19) and 11 general hospitals (out of 74). The participating hospitals were randomly selected from all acute care hospitals in the Netherlands. Inclusion criteria for the hospitals were: a minimum of 200 beds, an emergency department and an intensive care department. Specialty and psychiatric hospitals were not included. If a selected hospital had more than one location, all locations of the hospital were taken into account for patient record selection.

### Admission selection and record collection

A sample of 8400 admissions was selected: 4200 admissions of hospitalised patients and 4200 admissions of patients who died during the admission in 2004. Admissions with a diagnosis most related to obstetrics or psychiatry and admissions of children younger than 1 year old were excluded. The method and instruments were considered inappropriate for these medical specialties. Of each hospital a random sample of 200 admissions of patients discharged alive (admissions less than 1 day were excluded) and 200 admissions of deceased patients were selected with the hospital information system (inpatient database). For both patient groups 50 extra admissions were selected which could be used in case of missing patient files. One admission, the index admission, per patient was included. In order to assess the representativeness of the selected admissions per hospital, the distribution of gender, age, admission duration, most responsible specialty and diagnosis of the admissions were compared with the pattern for all admissions in the hospital in 2004.

Of the selected index admissions the medical and nursing records, and if available the outpatient records, were collected. Admissions without nursing or medical records were excluded in this study and reasons why records were missing were recorded. The composition of the records was not uniform in all participating hospitals. Some hospitals had all (medical, nursing and outpatient) records in one document archived in a central medical archive. Other hospitals had separate records archived on various departments. Outpatient records that were archived on multiple locations were not involved for logistic considerations. In two hospitals the records were scanned and displayed on a computer screen.

### Reviewer recruitment and training

The patient records were reviewed by a team of 66 nurses and 55 physicians. The team of recruited physicians consisted of 25 general internists, 20 general surgeons, 5 neurologists and 5 paediatricians. Most were recently retired. Recruitment of the physicians started through personal contacts of the project leader and was extended with contacts with the scientific association of internal medicine, surgery, neurology and paediatrics. The selection criteria for physicians were:

• at least ten years post graduate general clinical experience;

• good reputation among colleagues;

• no longer than five years retired;

• experience or affinity with analysis of incidents, complaints and errors;

• availability for at least one but preferably two days per week.

The nurses were recruited via the website of the association for nurses and websites of hospitals. The selection criteria for the nurses were:

• minimum five years clinical experience;

• experience or affinity with analysis of incidents, complaints and errors;

• availability for at least one, but preferably two days per week.

An additional expert panel of 18 physicians from other (sub)specialties was recruited to serve as experts for advices about accepted clinical practice. These specialists were authorities within their specialization and were recruited by the scientific associations of medical specialists. The panel consisted of specialists from all involved medical disciplines in the study. During the review process the physicians could get advice about accepted clinical practice from these authorities.

The nurses and physicians followed a one-day training in small groups (max 12 participants) led by one member of the research team and one experienced nurse or physician, respectively. During the training, the study protocol, definitions and review forms were explained and examples of (preventable) adverse events were discussed. The reviewers practiced with cases and the review forms and they were provided with a review manual. At the end of the training the nurses underwent a reliability test. After one month of reviewing, the reviewers had a half-day training session to discuss their problems concerning the review process and definitions and to update the reviewers with the latest insights about the review process. These training sessions were frequently repeated during data collection. The discussed problems were collected in a regularly updated Frequently Asked Questions (FAQ) document which was regularly handed out to all reviewers.

The reviewers were compensated for their review activities at an hourly rate and for expenses.

### Review process

**Figure 1:**
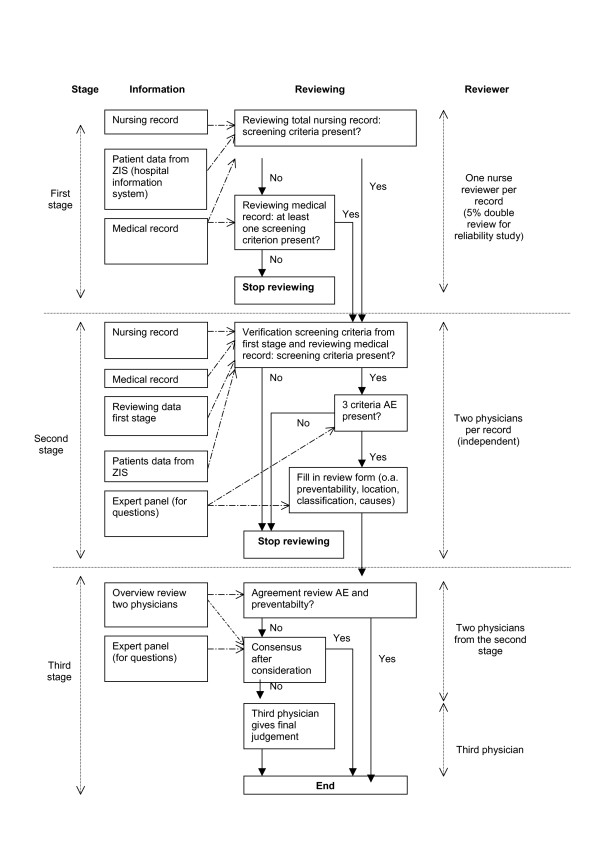
Diagram of the review process.

At each hospital, the patient records were reviewed in a three stage review process (Figure 1). In the first stage, nurses reviewed the complete nursing record from the selected index admission for the presence of one or more of 18 screening criteria known to be sensitive to the occurrence of an adverse event (table 3). If one or more screening criteria were found in the nursing record, the case was forwarded to the second stage of the review procedure. In case no screening criteria were found in the nursing record, the nurse also studied the medical record. The screening criteria in the records were marked with self-stick notes.

**Table 3:** Description screening criteria for potential adverse events [1]

**Screening criteria**
Unplanned admission before index admission (admission reasons are related to the index admission)
Unplanned readmission after discharge from index admission
Hospital-incurred patient injury (Permanent or temporary injury obtained (acquired) during index admission)
Adverse drug reaction
Unplanned transfer from general care to (an) intensive care (unit)
Unplanned transfer to another acute care hospital (after unexpected deterioration of the patient)
Unplanned return to the operating room
Unplanned removal, injury or repair of organ during surgery
Hospital-acquired infection or sepsis
Other patient complication
Development of neurological deficit not present on admission
Unexpected death
Cardiac or respiratory arrest
Injury related to abortion or delivery
Inappropriate discharge to home
Dissatisfaction with care documented in the medical record
Documentation or correspondence indicating litigation
Any other undesirable outcome not covered above

The records with one or more screening criteria were reviewed by two physicians of the same specialty (general internists, general surgeons, neurologists or paediatricians) in the second stage of the review process. They reviewed the records independently and they determined whether an adverse event had occurred and whether the adverse event had been preventable with an extended second stage review form. If the physician determined an adverse event, the review was continued with questions about the nature and impact of the adverse event; location and involved specialty; classification, preventability and causes of the adverse event.

If there was disagreement about the presence of an adverse event and/or preventability score between the two independent physicians, they started a consensus procedure (stage 3). In this consensus procedure the physicians considered and discussed both reviews and reconsidered their reviews in order to come to consensus. When there was still no agreement, a third trained reviewer gave a final judgement based on his own judgement and information of the other two reviewers.

During the second and third stage of the review process the physicians could ask advice from the 18 specialists in the expert panel about accepted clinical practice outside their professional competence, in order to improve their own judgement of the presence of adverse events and their preventability.

### Electronic review forms

The stage 1 and stage 2 review forms were paper based instruments converted into electronic templates in a highly secured web-based program called ProMIse [20]. In the hospitals, the reviewers filled in the electronic review forms with a computer connected to internet. Beforehand, general characteristics of all selected index admissions and patients were entered in the database. With a protected internet connection, the sampled data from the record review entered into ProMIse were immediately transferred and stored in a central database. No additional software was required. This way, no patient data on paper or portable electronic devices were left in the hospitals. In addition, working with electronic forms improved the efficiency of the data collection process and facilitated quality checks during data collection. The reviewers were trained to use this web-based program and received a manual for working with ProMIse. A helpdesk for technical problems was continuously available. The review forms on paper (in Dutch) are available from the author.

### Premises in participating hospitals

Some preparations were necessary before starting the study in the hospital. Authorisation of the governing board and the medical staff was a first condition. Further, selection of a random sample of patient admissions in 2004 from the hospital information system had to be performed by a hospital employee in cooperation with a member of the research team. After the sample had proven to be representative of all hospital admissions, employees of the archive departments started searching for the records. In some hospitals, laboratory and radiology results were stored separately in an electronic database to which access had to be arranged. During the review period a minimum of two computers with internet connection were needed in a separate room in the hospital that could be locked. A member of the research team managed the review process and arranged the records in the hospitals.

### Reliability study

To assess the variation of the review process between reviewers, a random sample of 5% of the records were independently reviewed by two nurses in the first stage. In the second stage 120 records were independently reviewed by a second pair of physicians.

### Confidentiality (Privacy)

In this study anonymity of hospitals, health care providers and patients was of utmost importance. Several measures were taken to ensure confidentiality of the collected information. Reviewers and researchers (study staff) signed a confidentiality agreement to maintain the secrecy of the information. The reviewers never reviewed in hospitals where they have ever been employed in the medical or nursing staff and the reviewers would never contact individual patients or physicians. During the data collection, the records were never left unattended and they were stored in a locked room.

Each admission received an unique study number so that patients' identity would not be revealed. Patient identifiers were kept in a dataset separately from the primary database. During the review process in hospital, the data were directly entered into a protected electronic database ProMIse. The reviewers had a personal password for the electronic database. The web-based database complied with the safety and privacy requirements. Patients' names were not included in the database and after completion of the data collection and analysis, medical record identifiers were destroyed. The identities of patients or physicians would not be revealed in research reports.

If a reviewer had any concerns during the review process about unrecognized potential deliberate harmful acts, illegal acts, or repetitive negligent behaviour, these concerns could be discussed with the ethics committee set up for this study.

### Ethical approval

The project and methods had been granted ethical approval by VU University Medical Centre in Amsterdam. The participating hospitals had formally consented to participate.

## Data Analysis

### Outcome measurements

The determination of adverse events was based on three criteria (table 4). To determine whether the injury was caused by health care a 6-point scale was used. Causation scores of 4–6 were classified as adverse events. From each adverse event found in this study the degree of preventability on a 6-point scale was measured and location, involved speciality, involved healthcare providers, classification and causes were registered. Furthermore, patient demographics such as age, sex, and social economic status (obtained by postal code) and admission characteristics like length of stay, admission status (elective, urgent, transfer or readmission), admission and discharge diagnosis, admission specialty, discharge status (dead, alive and discharge to home, home with outpatient care etc), and procedures were collected. Most of the patient and admission characteristics were provided by the participating hospitals from their hospital information system (inpatient database). In addition, for the patients who died during the index admission, the expected life time of the patient, should the adverse event not have occurred, was estimated by medical reviewers. The adequacy and completeness of the documentation of each studied record were judged by the reviewers.

**Table 4:** Outcome measurements [1,11]

Determination of the presence of an **adverse event **was based on three criteria:
1. an unintended (physical and/or mental) **injury **which
2. results in temporary or permanent **disability**, death or prolongation of hospital stay, and is
3. **caused **by health care management rather than the patient's disease
To determine whether the injury was **caused by health care management **or the disease process a 6-point scale was used:
1. (Virtually) no evidence for management causation
2. Slight to modest evidence of management causation
3. Management causation not likely (less than 50/50, but 'close call')
4. Management causation more likely (more than 50/50, but 'close call')
5. Moderate to strong evidence of management causation
6. (Virtually) certain evidence of management causation

The **degree of preventability **of the adverse events was measured on a 6-point scale, grouped into three categories:
*No preventability*
1. (Virtually) no evidence for preventability
*Low preventability*
2. Slight to modest evidence of preventability
3. Preventability not quite likely (less than 50/50, but 'close call')
*High preventability*
4. Preventability more than likely (more than 50/50, but 'close call')
5. Strong evidence of preventability
6. (Virtually) certain evidence of preventability

**Figure 2:**
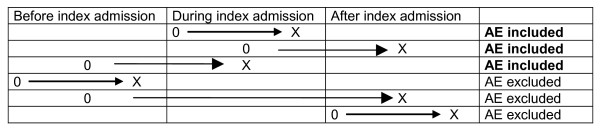
**Timing adverse event (occurrence and detection of AE)**. 0 = Occurrence AE, X = detection AE.

**Timing **of the adverse events in relation to index hospital admission. The **index hospital admission **was the admission sampled. Adverse events were recorded if they occurred during the index admission and that were detected during or after the index admission over the following 12-month period. Or adverse events that were related to hospital admissions within the 12-month preceding the index admission but were not detected until the index admission (Figure 2).

### Statistical analysis

During the data collection data checks (identify out-of-range answers, inconsistent responses and missing data) were performed on a regular basis. Data extracted from ProMIse were analysed using SPSS 12.0 for Windows.

The national weighted average incidence of adverse events in Dutch hospitals in categories of preventability was calculated, corrected for oversampling of university hospitals and of patients who died during hospital admission. Differences in adverse event rates between hospital types, discharge diagnoses and most responsible specialties were calculated using multi level analysis when appropriate, in order to disentangle variation at the patient and the hospital level. Potentially confounding determinants, such as age, sex, comorbidity, life-expectance and quality of the patient record, were identified and differences in adverse event rates between groups were adjusted for potentially confounding determinants using multilevel multivariate analysis. All incidence rates were calculated with 95% confidence intervals. For the subgroup of patients who died during admission, all analyses were replicated.

Direct medical costs associated with adverse events were measured as excess length of stay and charges for excess procedures during admission. For each admission in which the patient was discharged alive, the expected length of stay in hospital based on diagnosis, age and sex was estimated based on national data and excess length of stay was computed as the difference between actual length of stay and expected length of stay. Dutch guideline prices were used to value excess length of stay and procedures [21]. If not available, cost-prices or tariffs were used. Costs associated with adverse events were estimated using a linear regression model, adjusting for confounding factors such as age, sex and comorbidity when appropriate.

The inter-rater reliability of the review process by nurses and specialists was expressed as a kappa statistic with 95% confidence intervals and as percentage of records for which there was agreement. In the first stage the agreement between nurses was measured for finding screening criteria in the patient records. The kappa statistics in the second stage was measured for the determination of the degree of the injury, to what the degree the injury was caused by health care management and to what degree the adverse event was preventable.

## Discussion

### Strengths and limitations of this study

To address the need for empirical information regarding the epidemiology of poor quality and iatrogenic injury, the first large population-based retrospective medical record study was developed in New York in 1984 (Harvard Medical Practice Study, HMPS) [2]. The HMPS established a standard method by which adverse events are measured and it formed the basis of political discussions on patient safety in several countries [22]. This method was proven valid to identify adverse events and estimate their incidence in hospitals nation wide [17]. The HMPS method has been used nation wide in Australia, the UK, New Zealand, and Canada and has become the benchmark method for research on adverse events in hospitals and for assessing the status of patient safety in hospitals around the world [22]. Based on the results of the large studies of patient records, areas with problematic patient safety can be identified and specific patient safety actions can be implemented. In short, the strengths of this method are: effective for estimating adverse event incidence; almost no workload for hospital staff; no inconvenience for departments or interruptions of the health care process, and the data collection is easy to plan [6]. By using a highly similar protocol and instruments in our study it is possible to compare our results with those from previous (European) studies. With more than 8000 patient records, the Dutch study on the occurrence of adverse events in hospitals is the largest retrospective patient record study in Europe.

Although the results of these studies showed that the instruments are sensitive for identifying adverse events [17], some aspects can lead to an under- or overestimation of the adverse event incident rate. The record review method for identifying adverse events relies exclusively on data from hospital records. Only events documented in hospital records are included in the analysis and available information of events in the records can be insufficient for the adverse event determination [11,23] Without complete follow-up information on the patient, absolutely accurate estimates of disability are not possible [2]. This may underestimate the rate of adverse events in hospitals. In our study, the nurses and physicians assessed the completeness and adequacy of the records; also the relation between the quality of the information in the records and adverse events rate will be analysed. Insufficient records were excluded from the study.

Another potential source of error is missing records. In the HMPS the rate of adverse events in the missing records was measured by means of a follow-up study. The rates of adverse events and negligence in the follow up study were lower overall than in the initial survey [2]. In our study, hospital records without nursing or medical record were excluded and reasons why records were missing were recorded. A follow-up study to assess the adverse event rate in the missing records was not possible because of practical reasons.

Adverse events revealed after discharge are captured if they result in readmission to the hospital. If the patient is not readmitted, the adverse event is not discovered unless it is recorded in the hospital's outpatient record. It is not possible to estimate the number of adverse events that will be missed, but most adverse events that cause major disability or have a substantial financial impact probably require hospitalisation [2]. Moreover, at some participating hospitals in our study, it was not feasible to collect outpatient records as they are stored in many different archives. The lack of the outpatient records in some hospitals will lead to an underestimation of the adverse events incidence or of the effect.

Previous studies showed that adverse events can be identified accurately from information in hospital records, however, such records may not provide evidence or insight into the specific cause of an adverse event. The record review method is not the most suitable instrument to get insight into the specific cause of an adverse event [23].

### Validity

The internal and external validity of the record review study depends on the representativeness of the selected admissions. A non-representative sample can lead to under- or overestimation. To ensure that all admissions from 2004 were involved in the selection procedure, the random samples of admissions in the participating hospitals were taken from the hospital information system rather than from records available in the archive department. For each hospital representativeness of the sample is verified by comparing general characteristics of selected admissions with those of all admissions of the participating hospitals. If the selection of admissions was not representative, a new random sample was selected. Before we extrapolated the results of our study to all Dutch hospitals the characteristics of the total sample of 8400 admissions were compared with those of all admissions of all hospitals in the Netherlands.

### Reliability

Unlike injuries in the workplace or motor-vehicle accidents, which usually occur in healthy people, medical injuries in the hospital generally occur in those who are already ill. Therefore, it is sometimes difficult to distinguish disabling injuries caused by medical interventions from those attributable to the illness for which the patient is being treated. It can also be difficult to distinguish between injuries resulting from errors and those that could not reasonably have been prevented [24]. Thus, even with the carefully structured review process, there may still be substantial variation in the judgments of physician reviewers. Reliability estimates on the assessment of adverse events were only moderate in previous studies; those relating to the degree of impairment attributable to the adverse event were even lower [22].

In our study, several efforts were made to reduce the inter reviewer variation of the review process. Like the Canadian protocol, experienced nurses and physicians were recruited. They received a standardized training and a manual in which the research protocol, instruments and definitions were defined. During the study, reviewer performance was monitored and they received personal feedback. The use of electronic review forms and data from hospital information systems also enhanced the efficiency of the study and the reliability of the measurements. The electronic review form ensured complete data entry and computerized hospital data of the patients were transported into the electric forms in advance. This improved the completeness and quality of the data collection.

On top of the efforts from the Canadian protocol several activities were undertaken in our study to enhance the accuracy of the reviews. During the research process the reviewers could discussed their problems concerning the review process, cases and definitions in regularly organised discussion meetings. The questions and discussed problems were noted in a regularly updated frequently asked questions list. The precision of the reviews also depends on the efficiency of the design. For that purpose, in our study the reviewers had to focus on their own expertise: nurses concentrated on the nursing records; physicians examined mainly the medical records and the self-stick notes of the nurses in the nursing records. Moreover, four different specialties were involved for the medical review instead of two: general internists, general surgeons, neurologists and paediatricians. Records with, for example, neurological screening criteria were reviewed in the second stage by two neurologists. And for questions about accepted clinical practice the physicians could consult an expert panel of medical specialists. In the second stage all records were reviewed by two physicians instead of one. The consensus procedure and involvement of a third reviewer (in case of disagreement after the consensus procedure) should lead to a more reliable measurement.

### Modifications of protocol and instruments

The protocol and instruments used in this record review study were adapted from the Canadian record review study [1]. As a result of the Dutch pilot study, some modifications were made in the protocol and the instruments for the Dutch Adverse event Study [16].

• Selection of hospital admissions

The Dutch study included patients older than one year and oversampled deceased patients. In the Canadian study only patients over 18 years old were included and there was no oversampling for deceased patients. For this reason paediatricians were involved in the medical review of the record review process. The oversampling of deceased patients enables us to determine better the incidence of preventable deaths. Moreover, the incidence of adverse events is expected to be higher in this group, which makes the study more efficient.

• Review instrument

In the review form of the second stage we have changed some components and added some questions. The classification categories of the types of adverse events were changed and the questions for the medication related adverse events were made more specific. To get more insight into the causes of the measured adverse events the questions about the contributing factors were more extended and structured according to the PRIMSA classification of causes of incidents [25]. In the section 'quality of the record' questions about autopsy were added. Brennan (1991) wrote that the judgments of physicians that an adverse event has led to death also require a note of caution. Many patients who died after an adverse event had very serious underlying diseases, and several surely had shortened life expectancies independent of their iatrogenic injury [2]. In our study, the number of days of life lost as a result of the adverse event in the case of a terminally ill person was estimated to study the relation between the occurrence of adverse events and life expectancy. To improve the efficiency and quality of the data collection, control and analysis, the collected patient information was transported to a central database with a web-based program.

The changes in the protocol and the instrument are focussed on the improvement of the quality, efficiency and reliability of the adverse events determination and their preventability. In spite of these changes, comparison of the results of this study with the results of previous studies is still possible.

## Competing interests

The author(s) declare that they have no competing interests.

## Authors' contributions

MZ wrote the manuscript, prepared the protocol and instruments of the study, and collected and analysed data.

MB contributed to the manuscript, is the general supervisor of the retrospective record study, contributed to the design and conception of the study, and coordinated the study.

CW is the general supervisor of the Dutch patient safety research program and research group, contributed to the design and conception of the study, and contributed to the manuscript.

RW has developed the review forms within the web-based program ProMIse, managed the acquisition of data, facilitated data control during and after data collection and contributed to the statistical data analysis.

PG has been involved in revising the article critically for important intellectual content.

GW contributed to the design and conception of the study and has been involved in revising the article critically for important intellectual content (former general supervisor of the Dutch patient safety research program and research group).

All authors gave their approval of the final version of the manuscript.

## Pre-publication history

The pre-publication history for this paper can be accessed here:


